# Preliminary evidence of high prevalence of cerebral microbleeds in astronauts with spaceflight experience

**DOI:** 10.3389/fphys.2024.1360353

**Published:** 2024-06-14

**Authors:** Ford Burles, Morgan Willson, Parker Townes, Allison Yang, Giuseppe Iaria

**Affiliations:** ^1^ Canadian Space Health Research Network, Calgary, AB, Canada; ^2^ Neurolab, Department of Psychology, University of Calgary, Calgary, AB, Canada; ^3^ Departments of Radiology and Clinical Neurosciences, Cumming School of Medicine, University of Calgary, Calgary, AB, Canada

**Keywords:** susceptibility weighted imaging (SWI), MRI, cerebral amyloid angiopathy (CAA), cerebrovascular, brain structure

## Abstract

Long-duration spaceflight poses a variety of health risks to astronauts, largely resulting from extended exposure to microgravity and radiation. Here, we assessed the prevalence and incidence of cerebral microbleeds in sixteen astronauts before and after a typical 6-month mission on board the International Space Station Cerebral microbleeds are microhemorrhages in the brain, which are typically interpreted as early evidence of small vessel disease and have been associated with cognitive impairment. We identified evidence of higher-than-expected microbleed prevalence in astronauts with prior spaceflight experience. However, we did not identify a statistically significant increase in microbleed burden up to 7 months after spaceflight. Altogether, these preliminary findings suggest that spaceflight exposure may increase microbleed burden, but this influence may be indirect or occur over time courses that exceed 1 year. For health monitoring purposes, it may be valuable to acquire neuroimaging data that are able to detect the occurrence of microbleeds in astronauts following their spaceflight missions.

## Introduction

Forthcoming manned lunar missions, as well as prospective manned missions to Mars, underscore the importance of a deep understanding of both the short and long-term effects of spaceflight on astronauts’s health and wellbeing. Two salient environmental features present in spaceflight, microgravity and radiation exposure, are both known to produce medically-relevant changes to astronaut health. For instance, microgravity-induced bone density losses (Stavnichuk et al., 2020), muscle atrophy (Comfort et al., 2021), neuro-ophthalmic damage (Lee et al., 2020), and radiation-related cancer risks (Azzam et al., 2012) are all well-known health risks astronauts face during and following a spaceflight.

In addition to these risks, both prolonged microgravity exposure and increased exposure to ionizing radiation can produce a suite of changes to cardiovascular structure and function. Microgravity produces a cephalic fluid shift, diminished postflight orthostatic tolerance, cardiac arrhythmias (Anzai et al., 2014), arterial stiffening (Hughson et al., 2016), and potentially increases in intracranial pressure (Lawley et al., 2017), among other effects (Demontis et al., 2017). Increased exposure to ionizing radiation has also been shown to increase the risk of cardiovascular disease, by exacerbating atherosclerotic processes (Koutroumpakis et al., 2022), producing structural damage to blood vessels (Kuzichkin et al., 2022) and exacerbating microgravity-related thrombosis risks (Marshall-Goebel et al., 2019).

More recently, researchers have identified cerebral microbleeds (CMBs) as potential indicators of damage resulting from the cerebrovascular risks associated with spaceflight (Hähnel, 2020; Miller et al., 2022). Cerebral microbleeds are microhemorrhages (<10 mm in diameter) in the brain, producing covert lesions, and are visible as small hypointense foci on T_2_*-weighted gradient-recalled echo (GRE) and similar MRI sequences (Puy et al., 2021). Cerebral microbleeds represent powerful markers to identify the type and magnitude of small vessel disease, and are associated with an increased risk of cognitive impairment (Poels et al., 2012), stroke (Akoudad et al., 2015), and mortality (Akoudad et al., 2013). Importantly, they are considered early disease markers, often appearing in otherwise asymptomatic individuals before evidence of more serious morbidity (Igase et al., 2009).

Scientific investigation of small vessel disease typically focuses on the presence, quantity, and spatial location of cerebral microbleeds. With respect to location, cerebral microbleeds are typically localised as either “lobar” or “non-lobar ” (Gregoire et al., 2009). Lobar microbleeds are located in one of the lobes of the cerebral cortex itself, including both the cortical grey matter as well as the adjacent subcortical white matter. Non-lobar microbleeds include both “deep” cerebral microbleeds (located in subcortical grey matter, i.e., the thalamus and basal ganglia, along with nearby white matter structures, e.g., the internal and external capsules and the corpus callosum) as well as “infratentorial” microbleeds located in the brainstem and cerebellum. Clinically, lobar and non-lobar cerebral microbleeds have been shown to have different primary underlying causes, with lobar microbleeds more related to Cerebral Amyloid Angiopathy (CAA), and non-lobar microbleeds more associated with hypertension and arteriosclerosis (Puy et al., 2021). There is also evidence that exposure to ionizing radiation, as present in many radiation therapies, such as those for cancer treatment, facilitates the development of cerebral microbleeds (Morrison et al., 2019).

Given the variety of risk factors present in spaceflight environments that may produce cerebrovascular damage, and the utility of cerebral microbleeds as early indicators of such damage, we set out to evaluate the presence of these biomarkers in a sample of astronauts before and after a typical mission onboard the International Space Station (ISS). In particular, we investigated if astronauts exhibit more cerebral microbleeds after spaceflight as compared to before spaceflight, and determined whether or not previous spaceflight exposure was associated with the presence of cerebral microbleeds cross-sectionally.

## Methods

### Participants

We collected MRI data from 16 astronauts (seven female, aged *M*(*SD*) 45.72 (5.70) years old at first assessment), of which six had previous spaceflight experience (with mission durations of *M*(*SD*) 113.13 (74.38) days). Of these six individuals with previous spaceflight experience, their previous missions occurred *M*(*SD*) 2812 (822) days (*i.e.*, averaging over 7 years) before our initial data collection. Data were collected at three time points, one before and two after typical missions onboard the ISS (lasting *M*(*SD*) 200.31 (44.01) days): the first data collection was performed about 7 months prior launch (*M*(*SD*) 213.63 (118.35) days), the second “early postflight” was performed about 2 weeks after landing (*M*(*SD*) 12.44 (1.82) days), and the third “late post flight” on 14 of the 16 subjects was performed about 7 months after landing (*M*(*SD*) 221.07 (44.58) days). This study was approved by the institutional review boards of NASA’s Johnson Space Center and the University of Calgary. All participants provided written informed consent, and NASA has reviewed this manuscript and ensured it is compliant with the privacy standards of the NASA Astronaut Office.

### MRI data collection

At each of the three time points, we collected susceptibility-weighted images (SWI) using a 32-channel head coil on a 3T Siemens Verio MRI (running Syngo B19). SWI sequences are commonly used to identity cerebral microbleeds, and are more sensitive than T2*-weighted acquisitions (Shams et al., 2015). This gradient echo sequence had a 20.9 ms echo time, 2.9 ms repetition time, 20° flip angle, a pixel bandwidth of 121 Hz, and an in-plane acceleration factor of 3. Derived Siemens SWI images were produced by the acquisition software. Derived SWI slices had an axial-plane resolution of 0.625 × 0.625 mm and a left-right FOV of 288 voxels, and an anterior-posterior FOV of 384 voxels, with 72 slices spaced 2 mm apart.

### Microbleed identification

To identify microbleeds from the SWI images, we utilized heterogeneous methods and three raters with varying degrees of proficiency. Our expert rater, MW, has 10 years of experience as a board certified neuroradiologist in clinical and academic practice. Student raters, PT and AY were naive to microbleed identification prior to this project. FB ensured that raters were blinded to any data identifiers and administered a two-step CMB identification procedure. The first step was intended to have raters identify candidate CMBs across the entire dataset, and the second step was to generate explicit confirmation on the absence or presence of deduplicated and unified candidates across different timepoints and all raters. For the first step, the expert rater performed microbleed identification utilizing exhaustive manual identification. Student raters utilized a semi automated approach (Bian et al., 2013; Morrison et al., 2018) in which CMB candidates are identified automatically, and each student rater then manually pruned candidates to remove what they believed were false positive identifications.

After the first step was completed, FB deduplicated microbleeds identified by the raters, and unified identified microbleeds across different timepoints. This required moving the collected SWI volumes at different time points into alignment with one another using a rigid body registration in *antspyx* version 0.3.8. To ensure the appearance or disappearance of microbleeds from time point to time point was not due to rater error, FB then presented each unique microbleed candidate identified by any rater alongside the same volume in other timepoints from the same subject, and asked raters to identify the presence or absence of the microbleed candidate at each timepoint. Candidates were presented to raters in axial 64 × 64 patches, and raters were able to view three slices above and below the candidate centroid. At this stage, raters were presented with 21 unique candidates across three timepoints, requiring them to explicitly affirm or deny the presence of a microbleed in 63 images. Expert rater MW and student rater PT positively flagged the same 51 candidates and negatively flagged the remaining 12, resulting in a Cohen’s Kappa of 1. Student rater AY positively flagged the same 51 candidates, as well as an additional 2, resulting in a Cohen’s Kappa of 0.89 between AY and the other raters. Reported results are majority consensus, which are equivalent to the expert rater’s judgement.

### Analyses

We used paired-samples *t*-tests to compare microbleed counts, as well as microbleed presence (a boolean version of the microbleed count) between each adjacent time point. Other factors of interest that may influence the presence or quantity of microbleeds, i.e., previous spaceflight experience and age at time of preflight testing, were assessed with independent samples *t*-tests and bivariate correlation, respectively. For context, literature-derived microbleed incidences were compared against our sample incidences utilizing binomial tests. Due to the well-known sensitivity differences between different MRI field strength (Stehling et al., 2008; Conijn et al., 2011) and acquisition parameters (i.e., T_2_* GRE vs. SWI) (Goos et al., 2011; Shams et al., 2015), we restricted our literature comparisons to the studies using similar acquisition paradigms (Yates et al., 2014), i.e., SWI data collected at 3T, and did not include comparisons with literature values derived from larger studies with different acquisition paradigms (Poels et al., 2010).

## Results

**FIGURE 1:**
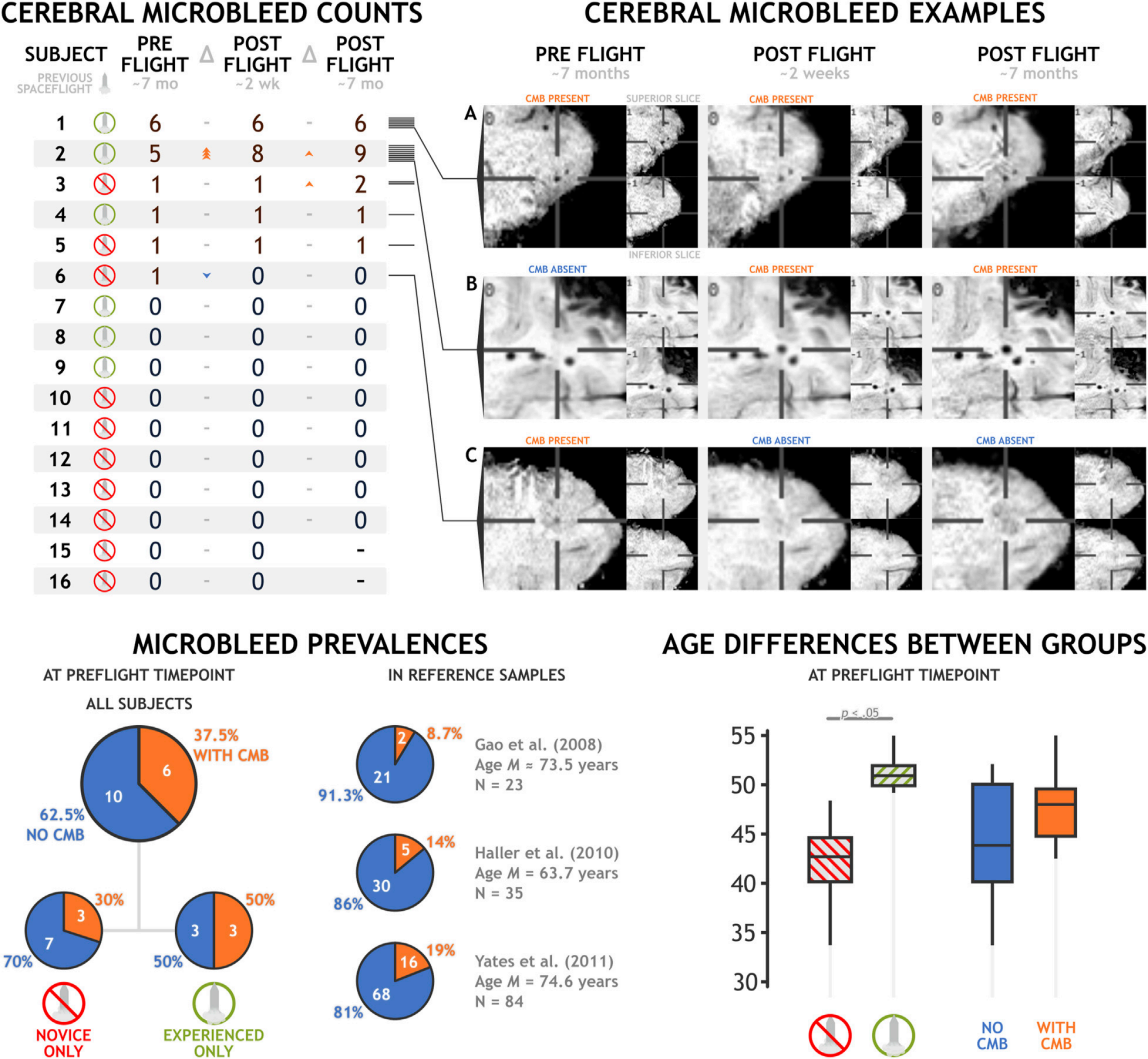
Cerebral microbleed counts and examples in a sample of 16 astronauts before and after a typical spaceflight mission. Exemplar **(A)** depicts a cerebral microbleed that remains present across preflight and postflight time points. **(B)** depicts a microbleed that manifested between the preflight and postflight time points. **(C)** depicts the only microbleed that appeared to resolve between preflight and postflight time points.

**FIGURE A1:**
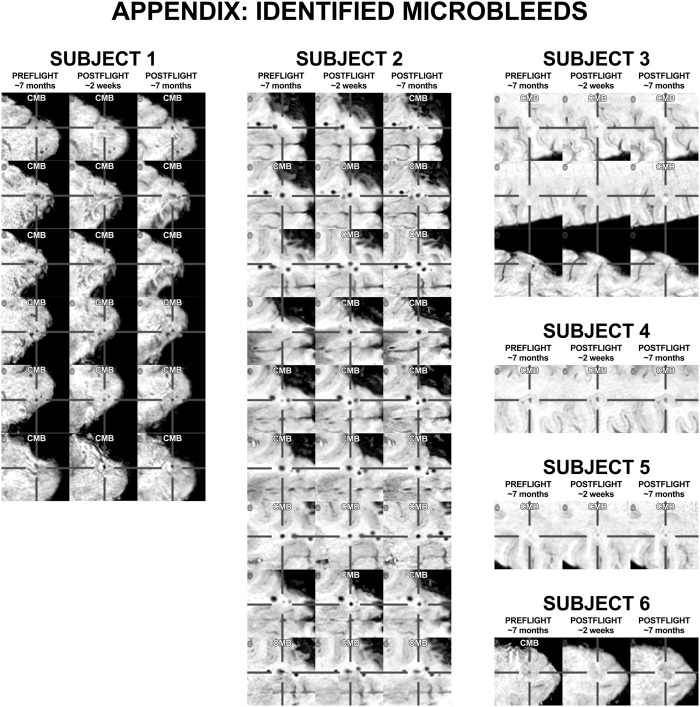
All positively identified microbleeds. Subject numbers correspond to those presented in Figure 1.

Microbleeds identified by majority consensus across our dataset are reported in Figure 1 and depicted in the Appendix Figure A1. We did not detect any microbleeds in the majority (i.e. 62.5%) of our participants at preflight timepoints. However we did detect 15 microbleeds in the remaining six (out of 16) participants at preflight, with individual counts ranging from a single to six microbleeds. At the early postflight time point, approximately 2 weeks after landing, we detected 17 microbleeds: three new microbleeds appearing in one participant and one microbleed in a different participant resolving. Finally, at our final time point, approximately 7 months after landing, we identified a total of 19 unique microbleeds, with novel microbleeds appearing in two subjects. All microbleeds identified in our dataset were lobar cerebral microbleeds; neither deep nor infratentorial microbleeds were detected. These lobar microbleeds were located most commonly in the frontal (60%) and temporal lobes (35%), with a single microbleed identified in the parietal lobe.

Generally, we did not detect any statistically significant increase in the presence or count of microbleeds after a typical stay onboard the ISS. Total microbleed count nominally increased from preflight to early postflight (Δ = 2, *t*_*15*_ = 0.620, *p* = .544, *d* = 0.155), and from early postflight to late postflight (Δ = 2, *t*_*15*_ = 1.472, *p* = .165, *d* = 0.393). We did not identify any instances of astronauts developing their first microbleed after spaceflight, and all novel microbleed identification was in individuals who exhibited microbleeds at our preflight assessment.

Astronauts with previous spaceflight experience demonstrated a nonsignificant trend towards being more likely to have a higher total microbleed count (*t*_*14*_ = 1.945, *p* = .072, *d* = 1.004), but no significant difference in simple microbleed presence (*t*_*14*_ = 0.764, *p* = .458, *d* = 0.524). However, astronauts with previous spaceflight experience were older than astronauts without previous experience (*MD* = 9.01 years, *t*_*14*_ = 4.827, *p* < .001, *d* = 2.493). Age is a known factor associated with an increased prevalence of cerebral microbleeds (Poels et al., 2010). In our dataset, astronaut age was not significantly associated with the presence (*r* = .304, *p* = .253) or amount (*r* = .297, *p* = .264) of microbleeds, but these effects trended in the directions expected by the previous literature. Reference prevalence estimates in healthy individuals using similar acquisition paradigms is limited. However, Yates and others (Yates et al., 2014) enumerated three studies that collected data in healthy controls utilizing SWI at 3T. These studies reported microbleed prevalences of 8.7% (Gao et al., 2008), 14% (Haller et al., 2010), and 19% (Yates et al., 2011) in samples of 23, 35, and 84 individuals, respectively. Mean group ages in these studies ranged from 63.7 to 74.6 years old, making them notably older than our astronaut group at a mean age of 45.72 years. Microbleed prevalence at our preflight time point in all sixteen astronaut participants, at 37.5%, trends higher than these three estimates (*p* = .002, *p* = .017, and *p* = .101, respectively). In the six astronauts with previous spaceflight experience (aged *M*(*SD*) 51.35 (2.089) years), microbleed prevalence at preflight timepoints was quite high, at 50%, trending above the literature estimates in older healthy samples (*p* = .011, *p* = .039, *p* = .087, respectively). The astronauts without previous spaceflight experience showed much lower prevalence, at 30%, a difference that did not significantly differ from literature values at this sample size (*p* = .050, *p* = .151, *p* = .414, respectively).

## Discussion

Astronauts are exposed to a variety of health threats during spaceflight. Here, we investigated the prevalence and incidence of cerebral microbleeds, small microhemorrhages indicative of cerebrovascular damage. We did not find strong evidence that spaceflight produced an increase in the incidence of cerebral microbleeds up to approximately 7 months after a typical mission onboard the ISS. We did, however, identify an increased prevalence of cerebral microbleeds in astronauts as compared to the non-astronauts samples reported in the literature, particularly in those astronauts with previous spaceflight experience. Interestingly, all 20 unique microbleeds that we identified were lobar grey and white matter bleeds, and we did not identify any deep or infratentorial microbleeds.

Strictly lobar microbleeds are a radiological feature typically associated with cerebral amyloid angiopathy (CAA), a cerebrovascular disease characterized by amyloid-β peptide deposition (Jung et al., 2020). CAA can be caused by the same amyloid protein that is associated with Alzheimer’s disease, but can also be present in individuals without a history of dementia (Cozza et al., 2023). The presence and quantity of cerebral microbleeds we have detected here is not direct and conclusive evidence of CAA, as conclusive diagnoses are typically done via autopsy (Charidimou et al., 2022). However, other research by Zu Eulenburg and others (Zu Eulenburg et al., 2021) in cosmonauts following typical missions onboard the ISS has identified increased levels of a handful of blood-based biomarkers of brain injury and neurodegeneration. These findings highlighted varying increases in levels of neurofilament light chain, tau, and Amyloid β 40 and 42 proteins at different points up to 3 weeks after cosmonauts returned from their missions. Zu Elenburg and others interpreted these findings as evidence of postflight reparatory processes following spaceflight-related brain injury.

An additional neurological feature associated with small vessel disease is the volume of perivascular spaces (PVS) - small fluid-filled regions adjacent to cerebral vasculature that facilitate fluid drainage and waste exchange (Wardlaw et al., 2020). Much like the presence of CMBs, enlarged PVS are considered markers of small vessel disease (Gyanwali et al., 2019). Recent studies in astronauts have identified that ISS missions were associated with an increase in PVS (Barisano et al., 2022), but astronauts with prior spaceflight experience appeared to be resilient to this effect (Hupfeld et al., 2022). In contrast to the observed pattern of cerebral microbleed prevalences, changes in PVS appear to be affected by spaceflight in a more acute manner, and most saliently in novice astronauts (Hupfeld et al., 2022). It is possible that both enlarged PVS and cerebral microbleeds are caused by a common feature of spaceflight, with PVS changes more acutely sensitive and the microbleeds manifesting later.

However, our findings did not reveal salient increases in the number of microbleeds between preflight and postflight in our sample, undermining the interpretation that spaceflight plays a causal role in increasing microbleed prevalence. It is possible that the postflight time frame of approximately 7 months was of insufficient duration for microbleeds to manifest after spaceflight exposure. For instance, a study investigating the time course of cerebral microbleed burden after radiation therapy found that microbleed count increased by 18% per year following treatment (Morrison et al., 2019). The highest microbleed burden reported in this study in an individual approximately 15 years after radiation therapy, suggesting the most salient microbleed burden should not be expected to follow immediately after radiation exposure, as an example of a mechanism that may be driving the effect we have observed. In our sample, astronauts with prior spaceflight experience landed from their last mission an average of over 7 years prior to testing, giving ample time for microbleeds to manifest. This process may not explain the presence of microbleeds in our participants without previous spaceflight experience, as their cumulative radiation exposure is likely lower than that of the astronauts with such experience. However, CMB incidence has been seen following exposure to other “extreme environments”, such as following high altitude cerebral edema (Kallenberg et al., 2008), and related markers of neurological damage may be present in air force pilots (Lim et al., 2012), all reinforcing the possibility that multiple causal factors may be driving these effects.

In conclusion, our study did not provide evidence of increased incidence of cerebral microbleeds up to 7 months following a low earth orbit spaceflight. However, we have identified preliminary evidence that prior spaceflight experience is associated with abnormally high cerebral microbleed prevalence. This is particularly concerning for astronaut health considering the fact that astronauts typically display strong “healthy participant” effects, and are generally expected to show lower morbidity and mortality than the general population (Reynolds and Day, 2019; Reynolds et al., 2021). It does, however, support previous researchers’ suggestions that spaceflight may produce neurological damage (Zu Eulenburg et al., 2021), and parallels the “rapid aging” paradigms supported by astronaut musculoskeletal degeneration (Vernikos and Schneider, 2010), as we observed microbleed burdens in otherwise healthy astronauts that met or exceeded those in healthy controls decades their senior. Future research will need to more clearly establish the prevalence, mechanisms, and time course of this potential cerebral microbleed burden in astronauts. As with many studies in astronaut populations, our sample size is small, and larger studies are needed to validate the effects we have reported to ensure they are not spurious or misattributed. Future work will also need to ensure that astronaut and comparison samples have similar ages, as spaceflight veterancy was confounded with age in our sample, preventing us from asserting a causal association between prior spaceflight experience and microbleed incidence. Unfortunately, NASA’s current Lifetime Surveillance of Astronaut Health Program MRI protocol does not include sequences appropriate for microbleed identification. Inclusion of an SWI (or manufacturer-equivalent), Quantitative Susceptibility Mapping (QSM), or a more innovative sequence (Sun et al., 2020) may be important to implement to monitor astronaut health and properly evaluate the cerebrovascular risk associated with spaceflight.

## Data Availability

The datasets presented in this article are not readily available to protect participant privacy. Requests to access secondary data should be directed to FB, cfburles@ucalgary.ca.
